# Techniques for Preserving the Nipple Areolar Complex in Chest Masculinization for All Chest Sizes

**DOI:** 10.7759/cureus.80757

**Published:** 2025-03-18

**Authors:** Tiffany Tse, Emery Potter, Kathleen Armstrong

**Affiliations:** 1 Plastic and Reconstructive Surgery, University of Toronto, Toronto, CAN; 2 Plastic, Reconstructive, and Aesthetic Surgery, Women's College Hospital, Toronto, CAN

**Keywords:** gender-affirming, gender-affirming surgery, gender confirmation surgery, masculinizing surgery, top surgery

## Abstract

Current literature on gender-affirming top surgery techniques predominantly focuses on achieving optimal binary male aesthetic outcomes. However, goals for surgery are unique, and preservation of the nipple-areolar complex (NAC) for aesthetics and sensation can be of primary importance to patients. This paper provides an algorithm for preserving the NAC based on chest size and native NAC position. Written consent was obtained for all before and after patient photos. Photos were taken as part of patient care and documentation from one surgeon at Women’s College Hospital in Toronto, Canada, between January 2020 and March 2022. Four techniques are highlighted in detail in this report: (1) keyhole (subcutaneous) mastectomies, (2) periareolar mastectomies, (3) nipple-preserving double-incision mastectomies, and (4) inverted T mastectomies.

Current literature for top surgery focuses primarily on double-incision mastectomies with free nipple grafts or a smaller subset of periareolar and keyhole mastectomies. We have outlined several techniques to preserve the NAC, and an algorithm has been recommended based on chest size and preoperative NAC position. NAC preservation is possible for most chest sizes when performing masculinizing chest surgeries. The algorithm we have described provides guidance for surgeons to choose which technique to use based on the patient’s breast volume, ptosis, and NAC position to preserve the entire NAC.

## Introduction

Chest masculinization surgeries, or "top surgeries," are procedures that remove breast tissue to match one’s gender identity with their body [1]. They are a gender-affirming surgery used to treat gender incongruence, distress, discomfort, or unease with the misalignment of physical appearance and gender identity [1]. Gender incongruence can be relieved with different treatments, including counseling, hormonal treatment, and gender-affirming surgeries [2]. Without undergoing chest masculinization surgery, many patients consistently bind their breasts with wraps and tight garments to conceal them, which can impact breathing and cause discomfort [3]. As such, for many transgender and gender-diverse patients, chest masculinization surgery can be an important element of their treatment, as distress over the presence of their breasts can contribute to many social, psychological, and physical problems [4].

Current literature on chest masculinization techniques predominantly focuses on achieving an optimal binary male aesthetic outcome [5]. However, preservation of the nipple-areolar complex (NAC) can significantly impact the physical appearance and psychological well-being of patients [6]. Patients may wish to preserve the NAC to (1) achieve a specific aesthetic outcome, (2) improve the likelihood of preserving NAC sensation and erectile function, and/or (3) they specifically fear caring for or losing the NAC grafts. As such, this paper provides a novel algorithm for preserving the NAC based on chest size and native NAC position to tailor outcomes based on patient preferences. Our paper highlights the four primary techniques used by a high-volume, single surgeon (greater than 350 masculinizing chest surgeries per year) to retain the native NAC including (1) keyhole (subcutaneous) mastectomies, (2) periareolar mastectomies, (3) nipple preserving double incision mastectomies, and (4) inverted T mastectomies. Transmen and nonbinary patients were recruited from a private outpatient plastic surgery clinic and Women’s College Hospital between January 2020 and March 2022. Preoperative and postoperative photos were taken with the patient’s written consent, and four patients were chosen to highlight the different mastectomy techniques. Each technique comes with its advantages and disadvantages, which will be outlined clearly in this technical report. Breasts that are smaller with no ptosis are suitable for a keyhole mastectomy [7]. Periareolar mastectomies are more appropriate for a slightly larger nonptotic breast with a larger areola [7]. In our experience, for breasts with lower pole volume but a good NAC position, nipple-preserving double-incision mastectomies are the best. Inverted T mastectomies work for individuals with larger breasts with ptosis.

As such, different methods can be used to preserve the NAC based on the patient’s chest and native NAC position. Each method should be tailored to the patient’s preferences and values for NAC preservation. This article was previously presented as a poster presentation at the Canadian Society of Plastic Surgeons Annual Meeting in June 2022.

## Technical report

Current literature on chest masculinization techniques focuses on achieving a flat, masculine-looking chest with minimal scarring and proper nipple positioning, what many believe to be the ideal surgical result [5]. Less consideration is given to preservation of the NAC, perhaps from an assumption that a standard masculine aesthetic outcome is the most important to patients. The most common top surgery, the double-incision mastectomy with free nipple grafting [8], can dramatically alter the aesthetic of the native NAC and typically does not allow for sensation preservation. In our experience, patients have different considerations for NAC preservation, including (1) maintaining erectile function; (2) fears associated with graft loss, healing, and final graft aesthetics; and (3) a desire to maintain the aesthetics of the native NAC. Thus, surgeons should tailor their approach to each patient’s preferences.

The following techniques were used by a single surgeon to preserve the NAC based on chest size and NAC position: (1) keyhole (subcutaneous), (2) periareolar, (3) nipple preserving double incision, and (4) inverted T mastectomies. Studies show that the anterior and lateral cutaneous branches of the second to sixth intercostal nerves are the primary sources of innervation to the breast skin and tissue, with the fourth and fifth intercostal nerves offering the most consistent source of innervation to the breast [9]. We have focused on preserving these nerves if the desire is to maintain nipple sensation or erectile function. The primary surgeon performed over 1700 top surgeries, of which approximately 62% are nipple-preserving double-incision (n = 1054), 16% are keyhole (n = 272), 16% are periareolar (n = 272), and 6% are inverted T mastectomies (n = 102). This paper is a technical report that highlights these four techniques used consistently by our primary surgeon.

Transmen and nonbinary patients that were assigned female at birth were recruited from a private outpatient plastic surgery clinic and Women’s College Hospital between January 2020 and March 2022. This study was reviewed and approved by the institutional ethics review board. Before and after photos were selected for four key patients who could demonstrate the techniques described, and written informed consent was obtained for all before and after patient photos. The mean age of the four patients was 23.5 (range, 23-24 years) and three of them identified as transmen, while one patient identified as nonbinary.

**Figure 1:**
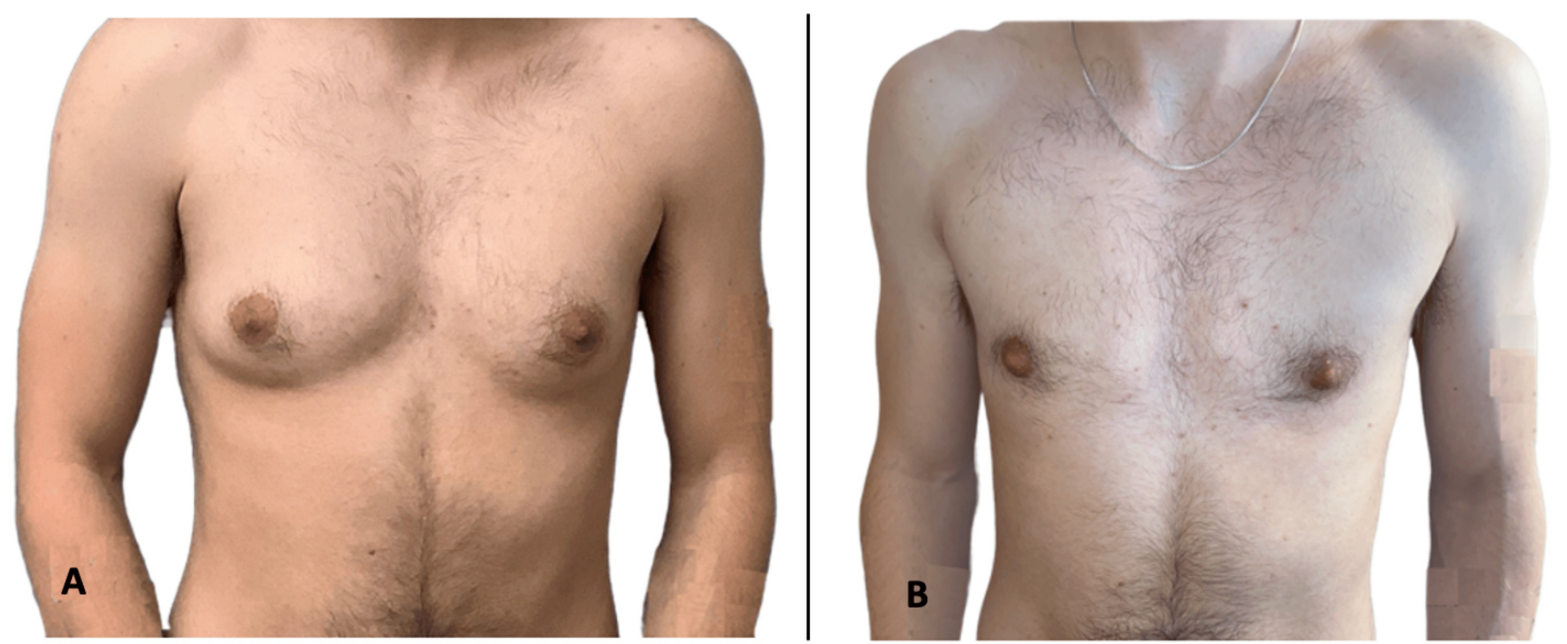
Patient one (A) Patient one preoperatively. (B) Patient one at three months postoperatively

Keyhole (subcutaneous) mastectomies are ideal for those who are small-chested with an acceptable NAC position. A small, semicircular, or half-moon incision is made on the lower border of the NAC, and the breast tissue is removed via tumescent liposuction and direct excision. The inframammary fold is obliterated. A drain is typically used and kept in for a week, placed through an inferior axillary incision. This technique does not resize the areola, and the NAC maintains its attachment to the superior chest. As the pedicled NAC is left untouched, nipple sensation can be preserved. The incision is typically half the length of the areola’s border, so it is barely visible and minimizes scarring. Figure 1 demonstrates patient one preoperatively and at three months postoperatively.

**Figure 2:**
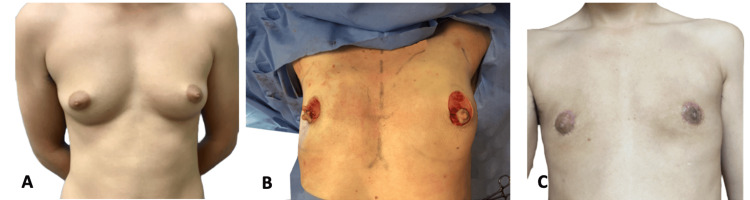
Patient two (A) Patient two preoperatively. (B) Patient two intraoperatively. (C) Patient two at 13 months postoperatively

In periareolar mastectomies, the breast tissue is removed through an incision along the inferior half of the NAC to preserve the dermal pedicle superiorly, similar to the keyhole technique. It is ideal for larger but nonptotic breasts with good skin quality that contracts to the chest wall. Some of the skin is removed via de-epithelization of a concentric circle around the NAC. This procedure removes the breast with tumescent liposuction and direct excision. A drawstring technique can be used to bring the skin together and to connect it to the edges of the areola, which can be resized and moved laterally if necessary. A ripple pattern may result immediately postoperatively, but this typically disappears in the weeks and months following surgery. The pedicled nipple-areolar complex is left intact, so some sensation can remain. The nipple can also be resized simultaneously with an inferior wedge technique. Figure 2 demonstrates patient two preoperatively, intraoperatively, and at 13 months postoperatively.

**Figure 3:**
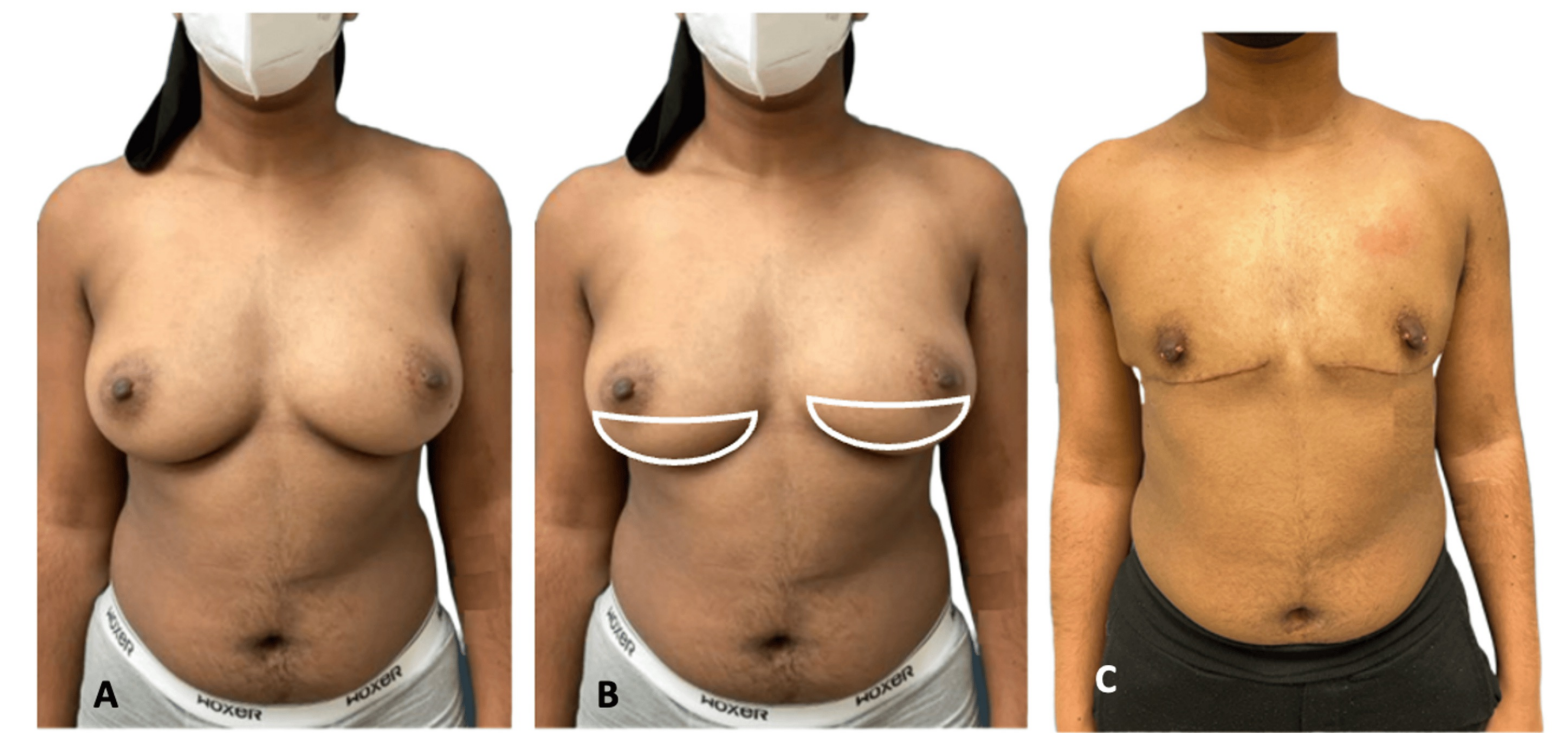
Patient three (A) Patient three preoperatively. (B) Patient three with wedge markings. (C) Patient three at six months postoperatively

In nipple-preserving double-incision mastectomies, an elliptical excision is made, similar to the double-incision mastectomy. However, the ellipse is performed 2 cm below the inferior border of the native NAC. This technique is appropriate for individuals with a larger breast size, good NAC position, and lower pole volume. The NAC is kept in its original position on the superior flap so that the areola cannot be moved or resized. Progressive tension sutures are used, like those used in double-incision mastectomies to avoid drains. Figure 3 demonstrates patient three preoperatively, with wedge markings, and at six months postoperatively.

**Figure 4:**
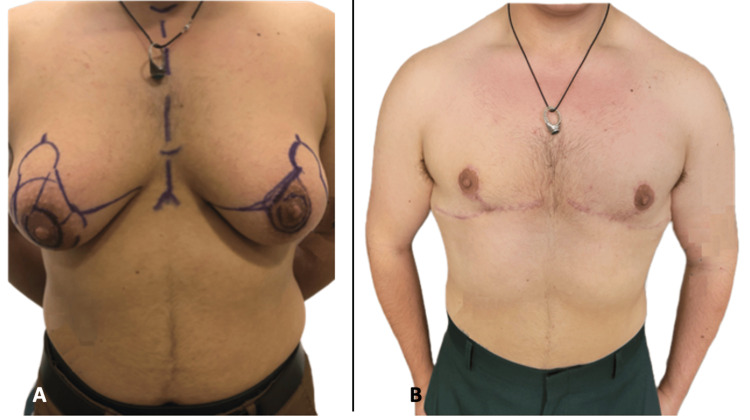
Patient four (A) Patient four preoperatively. (B) Patient four at seven months postoperatively

The inverted T mastectomy markings are similar to a Wise-pattern breast reduction but with different NAC position markings [10], a smaller mosque, and shorter vertical limb lengths. The NAC is positioned using key landmarks, including the lateral and inferior border of pectoralis major, sternal length, and chest width [10]. The mosque is approximately 5 cm wide and 4 cm long. The vertical limb lengths are 2.5-3 cm, so the NAC sits just above the inferior pectoralis border. This technique can be modified to allow for a more curved inframammary fold scar and a larger or more feminine centrally positioned NAC if such an aesthetic is desired. A circular incision is made around the new areola position, and a horizontal incision is performed at the level of the inframammary fold and lower border of the pectoralis. A vertical incision connects these two incisions, marking the appearance of an inverted T. Although the extra vertical incision leads to additional scarring when compared to double-incision mastectomies, it allows for NAC preservation through a superior medial dermal pedicle. NAC innervation is optimized, and nipple sensation can be preserved. The pedicle is 2 cm thick to avoid excess bulk behind the NAC. The dissections are carried out in a similar plane to the double-incision mastectomies, just above the breast capsule. Progressive tension sutures are used to avoid drains. The fourth patient demonstrates a more standard, masculine-appearing chest, with Figure 4 demonstrating patient four preoperatively and at seven months postoperatively. Inverted T mastectomies are ideal for medium to large-chested individuals, having been used to achieve a flat chest in individuals up to 715 grams. It can preserve NAC sensation. This patient reported a greater than 60% return of sensation at seven months postoperatively. A major limitation of the inverted T mastectomies is the flatness that can be achieved. Depending on the pedicle length, more or less bulk may be retained to keep the NAC attached. This is always explained to the patient.

Follow-up complications can be divided into major and minor complications. Examples of major complications experienced by patients include cosmetic revision, hematoma or seroma drainage in the main operating room, or an unplanned emergency visit. Examples of minor complications include cosmetic revision, hematoma or seroma drainage managed under local anesthetic, partial nipple or wound dehiscence, delayed wound healing, or an infection requiring antibiotics.

## Discussion

Chest dysphoria can be a major source of distress, and chest masculinization top surgery can offer benefits, including improvements in mood, confidence, and quality of life [11]. Although masculinizing chest wall reconstruction is increasing in prevalence [4], most of the current literature providing algorithms for chest masculinization surgery are based on binary masculine aesthetic outcomes and focus on double-incision mastectomies with free nipple grafts or a smaller subset of periareolar and keyhole mastectomies [12-16]. This paper provides surgeons with an algorithm for NAC preservation using four methods used by a single surgeon: the keyhole, periareolar, nipple-preserving double-incision, and inverted T mastectomies. Each technique has its advantages and disadvantages, which are summarized in Table 1:

**Table 1:** Advantages and disadvantages of each mastectomy technique

Technique	Advantages	Disadvantages
Keyhole	Smaller scar, possible nipple sensation preservation, less invasive	Smaller amount of breast tissue can be removed, no skin removed, no nipple repositioning
Periareolar	Smaller scar, possible nipple sensation preservation	Smaller amount of breast tissue can be removed, limited skin removal
Nipple-preserving double-incision	Can remove more breast tissue and redundant skin, good exposure	Areola cannot be moved or resized, larger scar
Inverted T	Can remove more breast tissue, good exposure, possible nipple sensation preservation	Larger scar, can be harder to achieve flat chest based on the pedicle length

**Figure 5:**
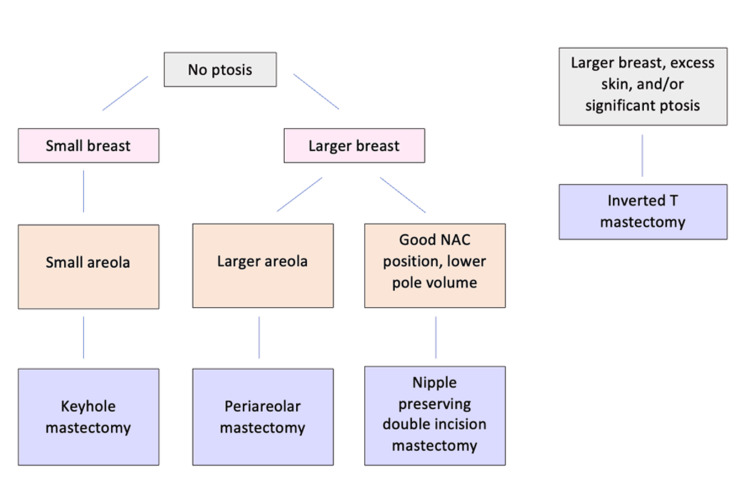
Algorithm for choosing a mastectomy technique to preserve nipple sensation based on chest type and size

In the senior author’s experience, keyhole mastectomies are an appropriate choice for smaller breasts with no ptosis and a small areola. Periareolar mastectomies are better for slightly larger, nonptotic breasts with larger areolas. A smaller amount of breast tissue can be removed using these two techniques, which our senior author has defined as up to approximately 300 grams if the patient has realistic goals and expectations regarding skin redundancy. Nipple-preserving double-incision mastectomies are suitable for breasts with lower pole volume and a good NAC position. Individuals with larger breasts, excess skin, and ptosis may benefit from an inverted T mastectomy. The nipple-sparing double-incision and inverted T mastectomies are less discussed in the literature; as such, we have described them in greater detail in this paper. Figure 5 is a flowchart with an algorithm that can be used to help distinguish when to use each technique for different chest types and sizes:

Our algorithm provides a standardized approach to help surgeons decide which mastectomy technique to use to preserve the NAC. Ultimately, the decision should be made after having a thorough conversation with the patient to understand their preferences and priorities. Limitations of this study include the small sample size and single surgeon experience. Additionally, patient satisfaction was not measured postoperatively in a standardized fashion. However, this surgeon has had extensive experience in chest masculinization surgery using these four techniques.

## Conclusions

Four main techniques can be used to preserve the NAC and avoid the use of nipple grafts. These techniques should be selected primarily based on patient preferences, which can vary, including priorities for scarring, flatness, NAC size, positioning preservation, and sensation. The literature currently describes many approaches to create a flat chest and to minimize scarring, but there is limited research focused on preserving the NAC for sensation and erectile function. As such, our report adds to the literature in providing surgeons with options to preserve the NAC and particularly highlights the nipple-sparing double-incision and inverted T mastectomies, which have been much less described recently. The next step would be to continue following up with these patients for their long-term outcomes with regard to their satisfaction based on their goals postoperatively. These surgeries should also be performed in a more diverse patient population, allowing for surgeons to tailor their approaches to different body types and preferences.

## References

[1] Ascha M, Sasson DC, Sood R (2022). Top surgery and chest dysphoria among transmasculine and nonbinary adolescents and young adults. JAMA Pediatr.

[2] Hadj-Moussa M, Ohl DA, Kuzon WM Jr (2018). Evaluation and treatment of gender dysphoria to prepare for gender confirmation surgery. Sex Med Rev.

[3] Pehlivanidis SG, Anderson JR (2024). A qualitative exploration of the motivations and implications of chest binding practices for transmasculine Australians. Int J Transgend Health.

[4] Agarwal CA, Scheefer MF, Wright LN, Walzer NK, Rivera A (2018). Quality of life improvement after chest wall masculinization in female-to-male transgender patients: a prospective study using the BREAST-Q and Body Uneasiness Test. J Plast Reconstr Aesthet Surg.

[5] Sayyed AA, Haffner Z, El Hawa AA (2022). Mutual understanding in the field of gender affirmation surgery: a systematic review of techniques and preferences for top surgery in nonbinary patients. Health Sci Rev.

[6] Moorefield AK, Stock A, Rose-Reneau Z, Singh PK, Azari Z, Wright BW, Singhal V (2021). Analysis of nipple-areola complex localization using male cadavers: Considerations for gender-affirming surgery. Aesthet Surg J Open Forum.

[7] Ammari T, Sluiter EC, Gast K, Kuzon WM Jr (2019). Female-to-male gender-affirming chest reconstruction surgery. Aesthet Surg J.

[8] Etemad SA, Furuyama WM, Winocour JS (2020). Double incision mastectomy with free nipple graft for masculinizing chest wall surgery. Plast Reconstr Surg Glob Open.

[9] Smeele HP, Bijkerk E, van Kuijk SM, Lataster A, van der Hulst RR, Tuinder SM (2022). Innervation of the female breast and nipple: a systematic review and meta-analysis of anatomical dissection studies. Plast Reconstr Surg.

[10] McEvenue G, Xu FZ, Cai R, McLean H (2017). Female-to-male gender affirming top surgery: a single surgeon’s 15-year retrospective review and treatment algorithm. Aesthet Surg J.

[11] Mehringer JE, Harrison JB, Quain KM, Shea JA, Hawkins LA, Dowshen NL (2021). Experience of chest dysphoria and masculinizing chest surgery in transmasculine youth. Pediatrics.

[12] Naides AI, Schultz JJ, Shulzhenko NO, Keith JD (2021). Chest masculinization technique and outcomes in 72 double-incision chest-contouring procedures with free nipple grafting. Plast Reconstr Surg Glob Open.

[13] Top H, Balta S (2017). Transsexual mastectomy: selection of appropriate technique according to breast characteristics. Balkan Med J.

[14] Morselli PG, Summo V, Pinto V, Fabbri E, Meriggiola MC (2019). Chest wall masculinization in female-to-male transsexuals: our treatment algorithm and life satisfaction questionnaire. Ann Plast Surg.

[15] Claes KE, D'Arpa S, Monstrey SJ (2018). Chest surgery for transgender and gender nonconforming individuals. Clin Plast Surg.

[16] Wolter A, Diedrichson J, Scholz T, Arens-Landwehr A, Liebau J (2015). Sexual reassignment surgery in female-to-male transsexuals: an algorithm for subcutaneous mastectomy. J Plast Reconstr Aesthet Surg.

